# Supplementary material for: Neighbourhood interactions drive overyielding in mixed-species tree communities
Source: Nat Commun. 2018 Mar 20;9:1144. doi: 10.1038/s41467-018-03529-w (PMC5861250; doi:10.1038/s41467-018-03529-w)
Supplement: Supplementary file 1 — Supplementary Information(PDF 1952 kb) [file 41467_2018_3529_MOESM1_ESM.pdf]

Supplementary Information

Neighbourhood interactions drive overyielding in mixed-species tree communities

Fichtner et al.

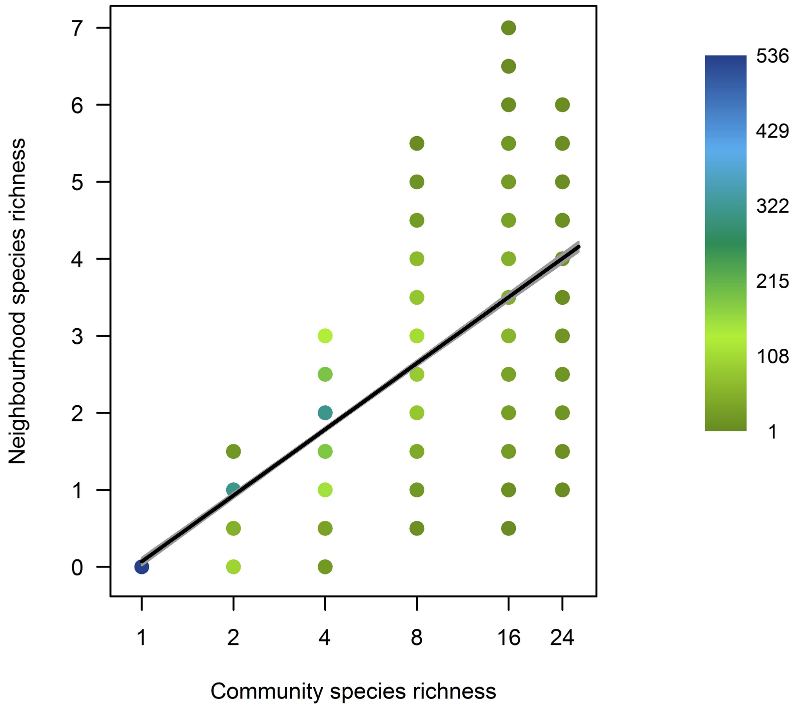

Supplementary Figure 1 | Relationship between tree species richness at the neighbourhood and community scale (log-transformed). The black line is a linear model fit (r^2: 0.66, P < 0.001), with shaded area representing the 95% confidence interval of the prediction. The colour of the points represent the number of observed values at site B (n_{total} =3018); x-axis is logarithmic. Note that neighbourhood species richness was calculated as the mean number of heterospecific neighbours in the study period (2011– 2016) to account for potential bias associated with differences in mortality (see Methods).

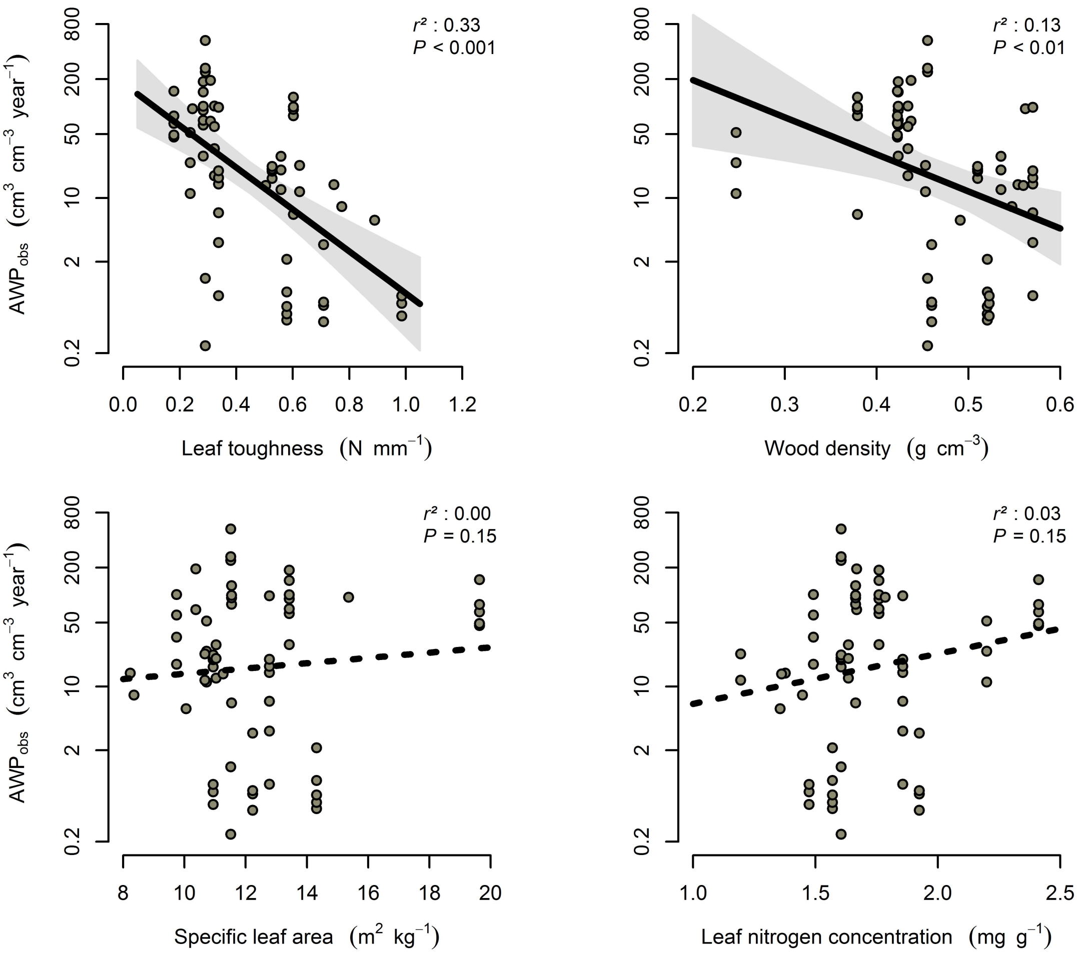

Supplementary Figure 2 | Univariate relationships between monoculture productivity and species' functional traits. Points represent observed values of standardised aboveground wood productivity (AWP_{obs}) of monocultures at site B. These four key traits are linked to productivity and shade tolerance, and thus reflect tree ecological strategies[1-3]. Solid lines represent significant ($P < 0.05$) and dotted lines non-significant ($P > 0.05$) relationships. The shaded area represents the 95% confidence interval. Y-axis is logarithmic.

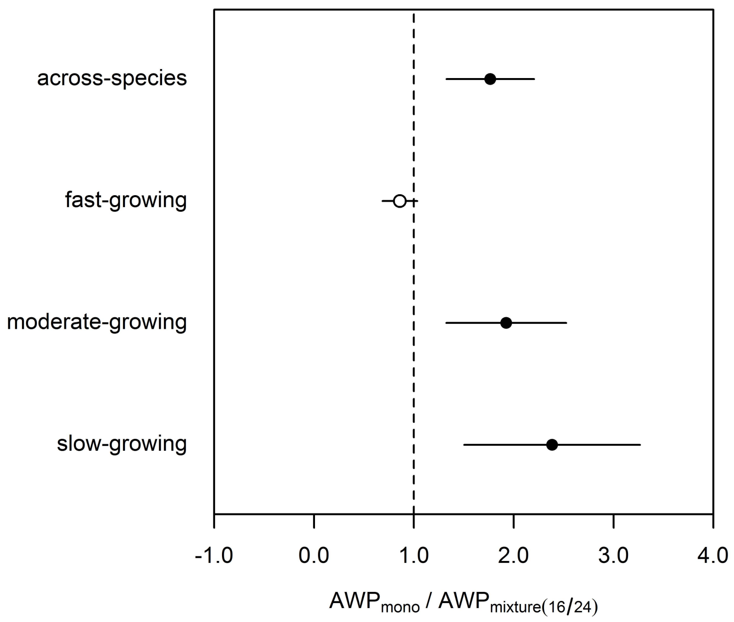

Supplementary Figure 3 | Proportional difference in observed species-specific standardised aboveground wood productivity (AWP_{obs}; productivity in 16- and 24-species mixtures divided by productivity in monoculture; site B) for slow, moderate- and fast-growing species. Slow-, moderate- and fast-growing species are defined as species in the 25%, 50% and 75% quantile of species-specific AWP_{obs} (cm^3 cm^{-3} $year^{-1}$) in monoculture, respectively. Points are means and error bars denote the 95% confidence intervals. Closed circles indicate significant ($P < 0.05$) and open circles indicate non-significant ($P > 0.05$) differences from 1. The dashed line indicates equivalent productivities in monoculture and mixture.

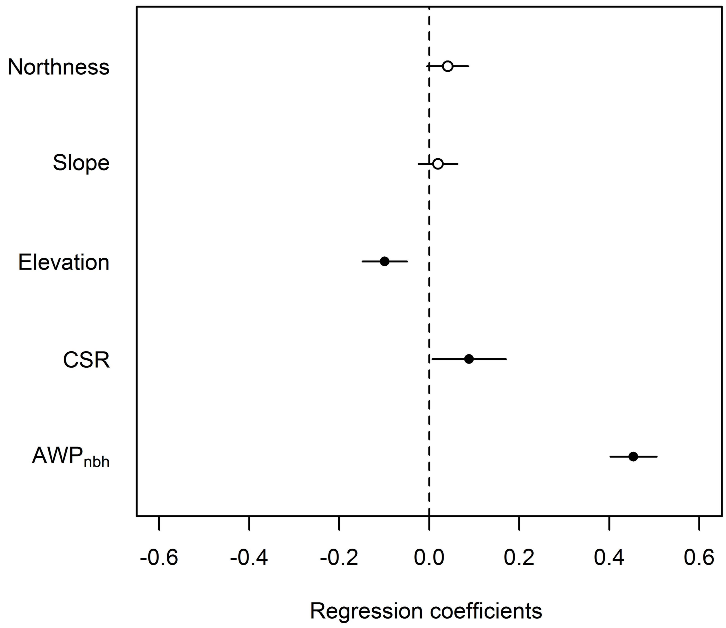

Supplementary Figure 4 | Regression coefficients of predictor effects on observed community aboveground wood productivity (AWP_{obs}). Community tree species richness (CSR, log_{10}-transformed), pedicted above-ground wood productivity based on neighbourhood interactions (AWP_{nbh}, log_{10}-transformed) and heterogeneity in topography (elevation, slope, 'northness'). Points are mixed-effects model fits for a global model containing all predictors (see Methods) and error bars denote the 95% confidence intervals. Closed circles indicate significant ($P < 0.05$) and open circles indicate non-significant ($P > 0.05$) effects. All predictors were standardised before analysis.

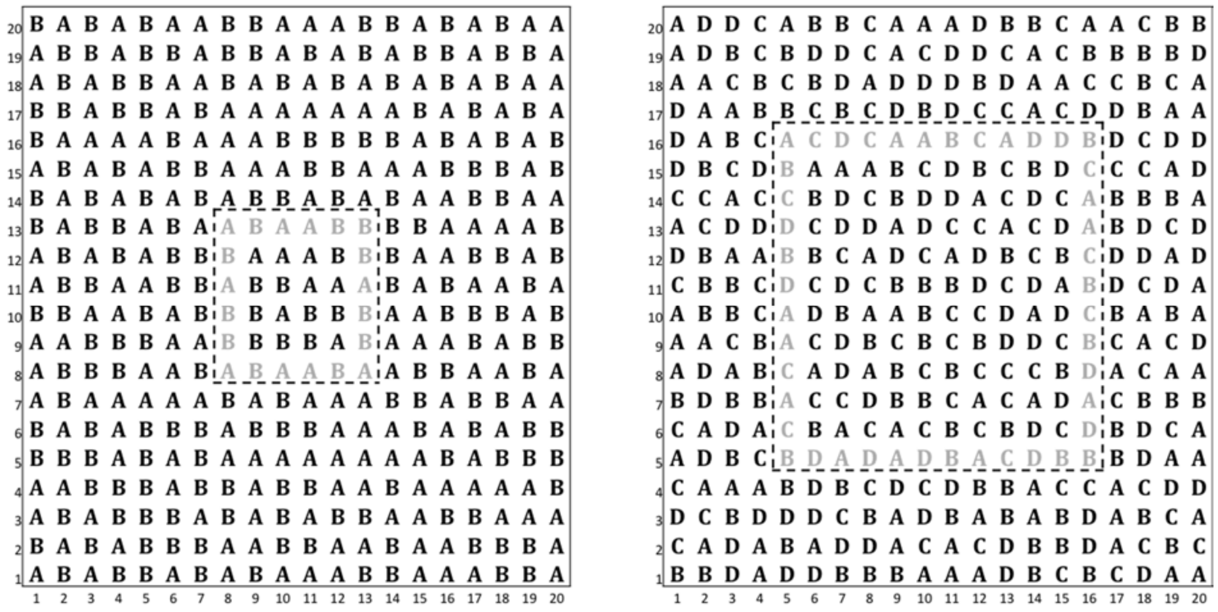

Supplementary Figure 5 | Focal tree sampling design. Schematic diagram illustrating the sampling areas for plots with monoculture or 2-species mixtures (left) and for plots with 4-, 8- , 16-, and 24-species mixtures (right). The alphabetic characters indicate different tree species that were randomly assigned to the plots. Tree individuals located within the dashed lines were used as focal trees (black letters), while trees of the outermost row could only act as neighbour-only trees (grey letters).The figure was taken from ref.[4].

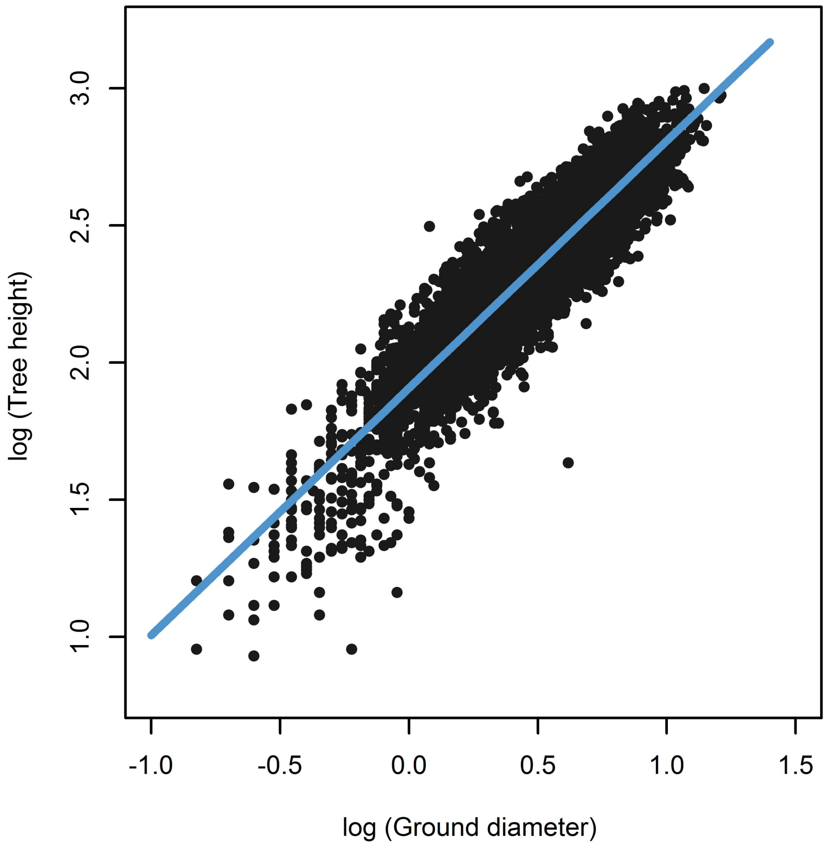

Supplementary Figure 6 | Relationship between mean ground diameter (cm) and tree height (cm) in the study period (2011–2016) of trees growing in the local neighbourhood of a focal tree. The blue line is a linear model fit (r^2: 0.83, $P < 0.001$) and points represent observed values. log = $\log_{10}$.

Supplementary Table 1 | Species pools of the two experimental study sites (site A and B) of the biodiversity experiment in subtropical China. Nomenclature: The Flora of China (http:// flora.huh.harvard.edu/china).

Species	Site
Acer davidii	A
Ailanthus altissima	B
Alniphyllum fortunei	B
Betula luminifera	B
Castanea henryi	A
Castanopsis carlesii	A
Castanopsis eyrei	A / B
Castanopsis fargesii	A / B
Castanopsis sclerophylla	A / B
Celtis biondi	B
Choerospondias axillaris	A
Cinnamomum camphora	A / B
Cyclobalanopsis glauca	A / B
Cyclobalanopsis myrsinifolia	A
Daphniphyllum oldhamii	A / B
Diospyros japonica	A / B
Elaeocarpus chinensis	B
Elaeocarpus glabripetalus	B
Elaeocarpus japonicus	B
Idesia polycarpa	B
Koelreuteria bipinnata	A
Liquidambar formosana	A
Lithocarpus glaber	A / B
Machilus grijsii	B
Machilus leptophylla	B
Machilus thunbergii	B
Manglietia fordiana	B
Melia azedarach	A
Meliosma flexuosa	B
Nyssa sinensis	A
Phoebe bournei	B
Quercus acutissima	A
Quercus fabri	A
Quercus phillyraeoides	B
Quercus serrata	A
Rhus chinensis	A
Sapindus saponaria	A
Schima superba	A / B
Triadica cochinchinensis	A
Triadica sebifera	A

Supplementary Table 2 | Regression coefficients estimates for the best-fitting neighbourhood model (site A). Estimates, standard errors (SE) and standard deviation (SD) were obtained from model with the lowest AIC score (see Supplementary Table 2). Note that the effect of CND in model 23 (Supplementary Table 6) was not significant (χ^2: 0.37, $P = 0.540$) and was therefore removed from the model with the lowest AIC. The variance explained by the fixed effects alone (marginal r^2) and by both the fixed and random effects (conditional r^2) was calculated according to ref.[5]. Error statistics include the mean absolute error ($MAE = \frac{1}{n}\sum_{i=1}^{n} |O_i - P_{i|}|^2$) and root mean squared error ($RMSE = \sqrt{\frac{1}{n}\sum_{i=1}^{n} (O_i - P_i)^2}$) based on fixed-effects estimates, where O are the observed and P are the predicted growth rates of the focal tree i and n is the number of observations[6]; log = $\log_{10}$. All predictors were standardised before analysis.

	Estimate	SE	*t*-value	*P*-value
Fixed effects				
Intercept	1.941	0.132	14.68	< 0.001
Initial focal tree volume (V, log)	0.536	0.037	14.45	< 0.001
Neighbourhood competition index (NCI, log+1)	-0.273	0.038	-7.24	< 0.001
Neighbourhood tree species richness (NSR)	0.229	0.074	3.11	0.002
V * NCI	0.064	0.015	4.37	< 0.001
V * NSR	-0.066	0.026	-2.55	0.011
NCI * NSR	-0.069	0.027	-2.58	0.010
V * NCI * NSR	0.026	0.010	2.68	0.007
Random effects				
SD (plot)	0.280			
SD (species identity)	0.440			
SD (neighbourhood species composition)	0.093			
SD (neighbour density)	0.046			
SD (residuals)	0.497			
Model fit				
r^2_m	0.48			
r^2_c	0.76			
MAE	0.54			
RMSE	0.69			

Supplementary Table 3 | Regression coefficients estimates for the best-fitting neighbourhood model of site A applied to site B data. See Supplementary Table 2 for explanation.

	Estimate	SE	*t*-value	*P*-value
Fixed effects				
Intercept	1.785	0.135	13.21	< 0.001
Initial focal tree volume (V, log)	0.511	0.035	14.61	< 0.001
Neighbourhood competition index (NCI, log+1)	-0.092	0.036	-2.55	0.011
Neighbourhood tree species richness (NSR)	0.216	0.061	3.57	< 0.001
V * NCI	-0.004	0.016	-0.24	0.813
V * NSR	-0.080	0.024	-3.32	0.001
NCI * NSR	-0.087	0.025	-3.51	< 0.001
V * NCI * NSR	0.040	0.010	3.87	< 0.001
Random effects				
SD (plot)	0.304			
SD (species identity)	0.521			
SD (neighbourhood species composition)	0.088			
SD (neighbour density)	0.041			
SD (residuals)	0.469			
Model fit				
r^2_m	0.32			
r^2_c	0.75			
MAE	0.55			
RMSE	0.70			

Supplementary Table 4 | Regression coefficients estimates for the best-fitting community model (site B). CSR: Community tree species composition; AWP_{nbh}: Predicted aboveground wood productivity based on neighbourhood interactions; Elevation: Mean elevation of a given plot within the study site. See Supplementary Table 2 for further explanations.

	Estimate	SE	*t*-value	*P*-value
Fixed effects				
Intercept	1.438	0.230	6.26	< 0.001
CSR (log)	0.090	0.042	2.18	0.032
AWP_{nbh} (log)	0.457	0.026	17.51	< 0.001
Elevation	-0.100	0.025	-4.00	< 0.001
Random effects				
SD (species composition)	0.310			
SD (residuals)	0.288			
Model fit				
r^2_m	0.57			
r^2_c	0.80			
MAE	0.32			
RMSE	0.44			

Supplementary Table 5 | Summary statistics of focal tree data. Initial (2011) and final (2016) focal tree size characteristics. Annual growth rates refer to a 5-year interval (2011– 2016), and neighbour characteristics refer to mean values in the study period. SD: standard deviation.

	Site A	Site B
	Mean (SD)	Mean (SD)
Initial tree wood volume (cm^3)	553.6 (1,319.8)	119.7 (219.9)
Initial tree height (cm)	129.5 (88.1)	86.7 (47.7)
Initial tree stem diameter (mm)	19.4 (14.5)	12.6 (8.1)
Final tree wood volume (cm^3)	11,324.7 (18,151.2)	8,572.6 (10,465.0)
Final tree height (cm)	419.2 (237.2)	359.2 (174.7)
Final tree stem diameter (mm)	58.1 (35.4)	59.1 (31.9)
Basal area of all neighbours (cm^2)	102.9 (73.5)	70.2 (52.1)
Basal area of larger neighbours (cm^2)	76.5 (71.1)	49.6 (50.1)
Growth rate - wood volume (cm^3 $year^{-1}$)	2,154.2 (3,461.9)	1,690.6 (2,070.4)
Growth rate -tree height (cm $year^{-1}$)	58.8 (36.6)	55.2 (29.0)
Growth rate - stem diameter (mm $year^{-1}$)	7.8 (5.5)	9.4 (5.5)
N° of species	24	24
N° of focal trees	3962	3018

Supplementary Table 6 | Model selection statistics for the neighbourhood model (site A). ΔAIC is the difference in AIC (Akaike Information Criterion) with respect to the best-fitting model (lowest value of AIC). The Akaike weight (w_i) is the relative likelihood of each model being the best-fitting model, given the complete set of candidate models[7]. The best-supported model according to the AIC and w_i is highlighted in bold. V: initial focal tree wood volume; NCI: neighbourhood competition index based on size-asymmetric competition; CND: conspecific neighbourhood density; NSR: neighbourhood tree species richness. Grey boxes indicate variable inclusion and models are ordered by increasing AIC values. * $P < 0.05$, ** $P < 0.01$, *** $P < 0.001$, n.s. $P > 0.05$.

	Fixed effects														
Model	V	NCI	CND	NSR	NSR * V	NSR * NCI	NSR * CND	V * NCI	V * CND	NCI * CND	NSR * V * NCI	NSR * V * CND	NSR * NCI * CND	ΔAIC	w_i
**23**	***	***	n.s.	**	*	**		***			**			**0.0**	**1.0**
24	■	■	■	■	■		■		■			■		94.3	0.0
12	■	■		■										97.8	0.0
19	■	■	■	■	■	■								98.5	0.0
16	■	■	■	■	■									98.6	0.0
15	■	■	■	■										99.4	0.0
5	■	■												99.5	0.0
22	■	■	■	■	■	■	■							100.1	0.0
20	■	■	■	■	■		■							100.4	0.0
17	■	■	■	■		■								100.7	0.0
11	■	■	■											101.1	0.0
18	■	■	■	■			■							101.2	0.0
21	■	■	■	■		■	■							102.4	0.0
25	■	■	■	■		■	■			■			■	103.7	0.0
1	■													222.9	0.0
6	■		■											223.1	0.0
7	■			■										223.3	0.0
13	■		■	■										224.7	0.0
14		■	■	■										2055.6	0.0
8		■	■											2057.9	0.0
2		■												2063.0	0.0
9		■		■										2064.7	0.0
4				■										2494.9	0.0
3			■											2496.1	0.0
10			■	■										2496.9	0.0

Supplementary Table 7 | Model selection statistics for neighbourhood models (site A) using neighbourhood species richness (NSR) or conspecific neighbour density (CND). See Supplementary Table 6 for further explanations.

Model	Fixed effects								
Model	V	NCI	NSR	NSR * V	NSR * NCI	V * NCI	NSR * V * NCI	ΔAIC	w_i
**15**	***	***	**	***	*	**	**	**0.0**	**0.50**
10	✓	✓	✓			✓		1.2	0.26
12	✓	✓	✓	✓		✓		3.2	0.10
13	✓	✓	✓		✓	✓		3.2	0.10
14	✓	✓	✓	✓	✓	✓		5.2	0.04
11	✓	✓	✓	✓	✓			98.6	0.00
8	✓	✓	✓	✓				98.7	0.00
7	✓	✓	✓					99.4	0.00
9	✓	✓	✓		✓			100.7	0.00
4	✓	✓						101.2	0.00
1	✓							224.5	0.00
5	✓		✓					224.9	0.00
2		✓						2064.6	0.00
6		✓	✓					2066.3	0.00
3			✓					2496.5	0.00

Model	V	NCI	NCD	NCD * V	NCD * NCI	V * NCI	NCD * V * NCI	ΔAIC	w_i
**10**	***	***	n.s.			***		**0.0**	**0.41**
12	✓	✓	✓	✓		✓		1.5	0.19
13	✓	✓	✓		✓	✓		1.9	0.16
15	✓	✓	✓	✓	✓	✓	✓	2.0	0.15
14	✓	✓	✓	✓	✓	✓		3.2	0.08
4	✓	✓						91.9	0.00
7	✓	✓	✓					93.5	0.00
9	✓	✓	✓		✓			95.3	0.00
8	✓	✓	✓	✓				95.4	0.00
11	✓	✓	✓	✓	✓			97.3	0.00
1	✓							215.3	0.00
5	✓		✓					215.4	0.00
6		✓	✓					2050.3	0.00
2		✓						2055.4	0.00
3			✓					2488.5	0.00

Supplementary Table 8 | Model selection statistics for neighbourhood models (site A) using a size-symmetric neighbourhood competition index (NCI). See Supplementary Table 6 for further explanations.

	Fixed effects														
Model	V	NCI	CND	NSR	NSR * V	NSR * NCI	NSR * CND	V * NCI	V * CND	NCI * CND	NSR * V * NCI	NSR * V * CND	NSR * NCI * CND	ΔAIC	w_i
**23**	***	***	n.s.	***	*	***		n.s.			*			**0.0**	**0.95**
25	■	■	■	■		■	■			■			■	8.6	0.01
19	■	■	■	■	■	■								9.0	0.01
17	■	■	■	■		■								9.4	0.01
22	■	■	■	■	■	■	■							10.3	0.01
21	■	■	■	■		■	■							10.7	0.00
12	■	■		■										14.1	0.00
24	■	■	■	■	■		■		■			■		14.7	0.00
5	■	■												15.4	0.00
15	■	■	■	■										16.1	0.00
16	■	■	■	■	■									16.1	0.00
11	■	■	■											16.4	0.00
20	■	■	■	■	■		■							17.1	0.00
18	■	■	■	■			■							17.1	0.00
1	■													33.7	0.00
6	■		■											33.8	0.00
7	■			■										34.0	0.00
13	■		■	■										35.5	0.00
2		■												2300.2	0.00
8		■	■											2300.9	0.00
9		■		■										2301.1	0.00
14		■	■	■										2302.7	0.00
4				■										2305.6	0.00
3			■											2306.8	0.00
10			■	■										2307.6	0.00

Supplementary Table 9 | Mean mortality rates of monocultures and species mixtures in the study period (site B). CSR: Community tree species richness. Data refer to the across species response within a species richness level based on focal and neighbour-only trees for the study period 2011–2016.

CSR	Mortality (%)
1	24.3
2	22.0
4	23.0
8	20.3
16	20.6
24	27.4

Supplementary References

1. Valladares, F. & Niinemets, Ü. Shade tolerance, a key plant feature of complex nature and consequences. *Annu. Rev. Ecol. Evol. Syst.* **39**, 237–57 (2008).

2. Lasky, J. R., Uriarte, M., Boukilic, V.K. & Chazdonc, R. L. Trait-mediated assembly processes predict successional changes in community diversity of tropical forests. *Proc. Natl. Acad. Sci. USA* **111**, 5616–5621 (2014).

3. Fichtner, A. *et al.* From competition to facilitation: how tree species respond to neighbourhood diversity. *Ecol. Lett.* **20**, 892–900 (2017).

4. Li, Y. *et al*. Site and neighborhood effects on growth of tree saplings in subtropical plantations (China). *For. Ecol. Manage.* **327**, 188–127 (2014).

5. Nakagawa, S. & Schielzeth, H. A. general and simple method for obtaining R2 from generalized linear mixed-effects models. *Methods Ecol. Evol.* **4**, 133–142 (2013).

6. Harrel, F. E. *Regression Modelling Strategies* (Springer, 2001).

7. Burnham, K. P. & Anderson, D. R. *Model selection and multimodel inference: a practical information – theoretic approach* (Springer, 2002).
